# Optical coherence tomography angiography parameters in Marfan syndrome: Genetic determinants and associations with cardiovascular manifestations

**DOI:** 10.1371/journal.pone.0347666

**Published:** 2026-04-24

**Authors:** Klaudia Kéki-Kovács, Roland Stengl, Bence Ágg, András Végh, Erika Maka, Mária Bausz, Csaba Bödör, Cristina M. Șulea, Máté Csonka, Kálmán Benke, Miklós Pólos, Zoltán Szabolcs, Zoltán Zsolt Nagy

**Affiliations:** 1 Department of Ophthalmology, Semmelweis University, Budapest, Hungary; 2 Heart and Vascular Center, Semmelweis University, Budapest, Hungary; 3 Hungarian Marfan Foundation, Budapest, Hungary; 4 Center for Pharmacology and Drug Research and Development, Department of Pharmacology and Pharmacotherapy, Semmelweis University, Budapest, Hungary; 5 Department of Pathology and Experimental Cancer Research, Semmelweis University, Budapest, Hungary; 6 Department of Cardiac Surgery, University of Halle, Germany; Cairo University Kasr Alainy Faculty of Medicine, EGYPT

## Abstract

In addition to aortic manifestations, Marfan syndrome can affect retinal vessels. Our aim was to evaluate retinal circulation, and its correlation with genotype and cardiovascular manifestation identifying predictors of aortic involvement (dilation and/or dissection). In the retrospective, cross-sectional study, 39 Marfan syndrome patients with optical coherence tomography angiography records were included. Retinal thickness, superficial and deep vessel density in total retina, fovea, parafovea, perifovea, area and perimeter of foveal avascular zone, fractal dimension were measured. Two groups were created by mutation type: haploinsufficient, dominant negative. Latter were divided into two subgroups according to whether mutation resulted in cysteine elimination. Subjects were assigned into cardiovascular risk based on previous aortic surgery. Retina of haploinsufficient patients was thinner in total, foveal, parafoveal, perifoveal areas compared to dominant negative subjects (p ≤ 0.047). Retinal thickness of haploinsufficient individuals was thinner in total, parafoveal areas compared to dominant negative without (LSD p = 0.038, Bonferroni p = 0.027, respectively) and with cysteine elimination variants (LSD p = 0.032, Bonferroni p = 0.002, respectively). In fovea and perifovea, retinal thickness was decreased in haploinsufficient patients in comparison to dominant negative with cysteine elimination group (Bonferroni p ≤ 0.029). Total, parafoveal, perifoveal superficial and total, parafoveal deep vessel density of subjects who underwent aortic surgery were lower compared to non-operated patients (p ≤ 0.043). To the best of our knowledge, our study is the first to describe a relationship between genotype and optical coherence tomography angiography parameters in Marfan syndrome. These findings along with correlations between genetics and cardiovascular manifestations reported previously, suggest that these parameters may be indirect predictors of increased cardiovascular risk. Here we demonstrated associations between these parameters and aortic involvement.

## Introduction

Marfan syndrome (MFS) is a hereditary connective tissue disorder caused by mutations in the fibrillin-1 gene (*FBN1*), which encodes the fibrillin-1 protein [1]. While the syndrome is inherited in an autosomal dominant manner, about 25% of the cases develop sporadically due to *de novo* mutations [2,3]. The prevalence of MFS is estimated at 6.5–30 in 100.000 subjects [2,4,5] with no predilection for either sex [2]. The clinical phenotype is highly variable in severity and usually involves the cardiovascular, ocular, and musculoskeletal systems. The manifestations can be present in many forms, such as ascending aortic aneurysm and dissection, mitral valve prolapse, ectopia lentis (EL), and long bone overgrowth [6]. The most common and dangerous cardiovascular complication is aortic root dilation, which can lead to life-threatening aortic dissection if not treated in time. Approximately two-thirds of aortic dissections in MFS patients involve the ascending aorta (type A dissection). Compared to the mortality rate of surgical intervention for acute type A aortic dissection, which can be as high as 20%, the mortality of a prophylactic aortic surgery is about 1.5%, highlighting the relevance of prevention [7]. The main indication for preventive surgery is the diameter of the aorta, with a threshold of 50 mm in patients with MFS. In the presence of any risk factors, this value can be as low as 45 mm. However, acute vascular events can occur even below this size, so it is necessary to identify predictors that could help optimize the indication and timing of prophylactic surgery [7,8]. Therefore, in addition to timely recognition of MFS, special attention must be paid to cardiovascular involvement.

Mutations in *FBN1* may result in two different effects: dominant negative (DN) and haploinsufficient (HI). The HI effect leads to degradation by the nonsense-mediated decay system of the mutant mRNA (mainly frameshift, nonsense, and splicing variants). In the case of DN variants, mutant fibrillin-1 monomers are produced, resulting in disrupted polymerization and structurally damaged microfibrils (mainly missense and in-frame mutations) [9,10]. DN mutations, especially those related to cysteine elimination or formation, are associated with a higher prevalence of EL [11,12], whereas patients carrying HI variants are more likely to have a more severe cardiovascular and musculoskeletal presentation [7,13]. Despite the increasing attention in recent years to the qualitative approach to genetic testing of *FBN1* variants, the focus has been mainly on cardiovascular complications. Genotype-phenotype associations of ocular manifestations are poorly understood.

According to a study conducted on a large and diverse cohort of MFS patients, subjects with ocular symptoms were nearly twice as likely to have aortic aneurysms or dissections compared to MFS patients without ocular manifestations. This association was almost as strong for mitral valve prolapse and tricuspid valve abnormalities. Patients with ocular symptoms were more likely to have cerebral aneurysms and dissections, pulmonary artery aneurysms, and involvement of other major arteries. Retinal detachment or tear showed an even stronger association with cardiovascular comorbidities, more than doubling the risk of aortic aneurysm or dissection and tripling the risk of arrhythmias, tricuspid valve abnormalities, and coronary artery aneurysm and dissection [14].

Previously, several studies have described that MFS is associated with increased arterial tortuosity of the vertebral arteries and the aorta [15,16]. Our group was the first to recognize increased arterial tortuosity in visceral arteries (splenic and renal) in MFS patients compared to the non-MFS population, the course of which is less affected by skeletal deformities. This increased tortuosity could be linked to more severe aortic manifestations [16,17], making it a promising predictor of aortic involvement severity. Furthermore, the above findings indicate that, in addition to the aorta, other vessels may also be affected by the syndrome. For these reasons, we decided to investigate the retinal circulation, due to its accessibility and utility in clinical practice. The retina is an easily accessible end organ that resembles cerebral and coronary circulation in many vascular, structural, and physiological characteristics. Examination of the retina could thus provide an excellent opportunity to evaluate microvascular circulation [18].

The examination of retinal blood vessels and blood flow provides valuable information about the state of the microvasculature and thus could contribute to the early diagnosis and follow-up of diseases affecting the systemic circulation, such as MFS. This may have many advantages, primarily the prevention of life-threatening cardiovascular complications. Therefore, our goal was to evaluate retinal circulation using optical coherence tomography angiography (OCTA) parameters and to investigate its relationship with genotype and cardiovascular involvement in MFS.

## Materials and methods

### Studied population

In this retrospective cross-sectional, single-center study, 128 eyes of 64 genetically confirmed MFS patients were examined. Subjects were recruited at the Marfan outpatient clinic of the Heart and Vascular Center of Semmelweis University, Budapest, Hungary, and underwent ophthalmological examinations at the Department of Ophthalmology, Semmelweis University, Budapest, Hungary between April 15, 2021, and April 30, 2023. The research was approved by the Semmelweis University Regional and Institutional Scientific and Research Ethics Committee (SE RKEB 89–1/2021). Genetic test results were gathered by a retrospective analysis of data collected in the framework of the research project “Genetic database of individuals with Marfan syndrome” (ethical license number ETT TUKEB 12751-3/2017/EKU and 6475-7/2021/EÜIG).

Participants (parents or legal guardians in case of subjects under 18 years of age) gave written informed consent to participate in the study. The subjects were examined in accordance with the principles of the Declaration of Helsinki. During data collection, the authors were able to identify which patients the data belonged to, which was necessary to exclude recordings of inadequate quality from the study. After selecting the appropriate recordings, personal data was rendered unrecognizable so as not to influence further evaluation.

**Fig 1:**
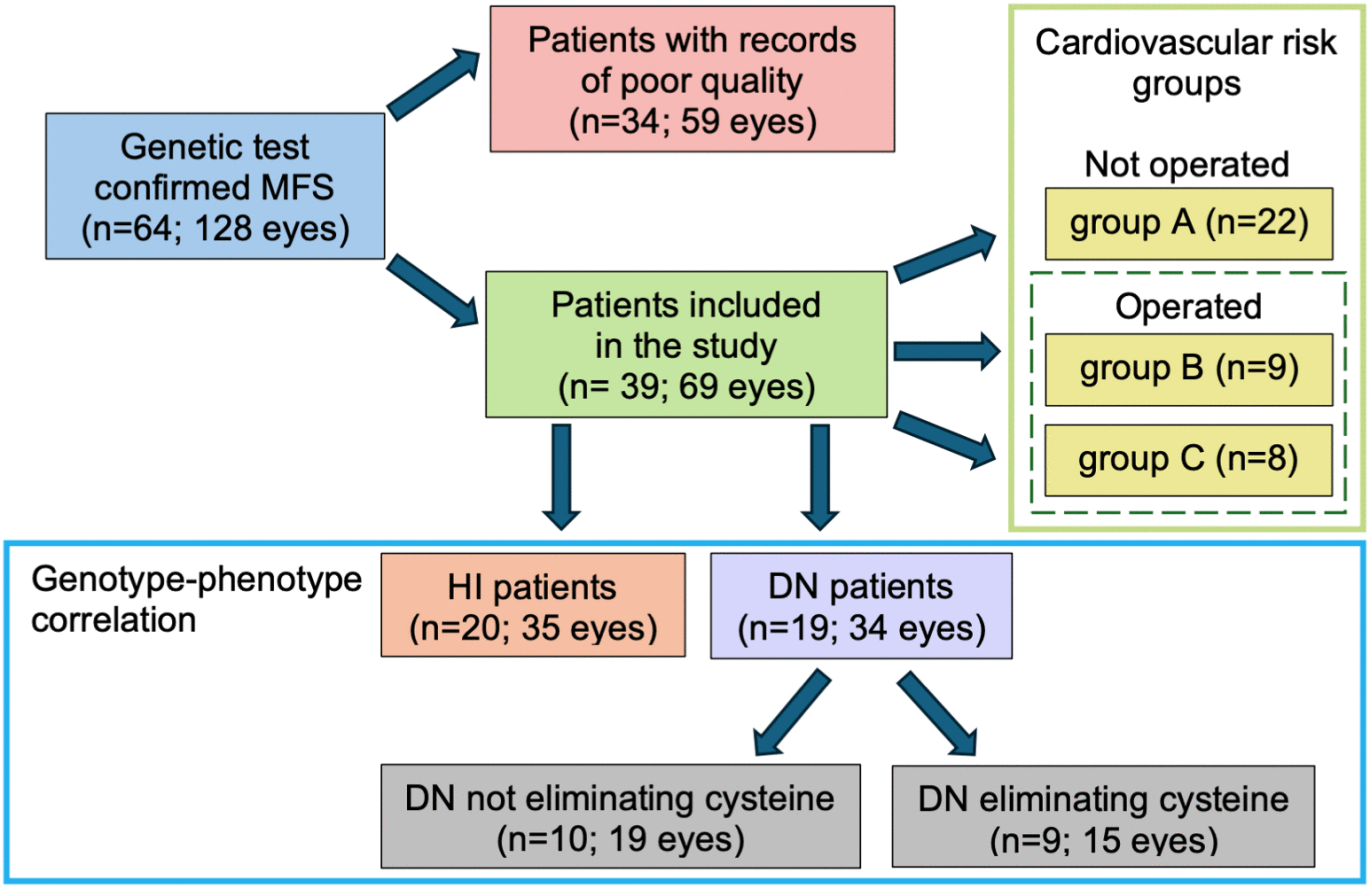
Flowchart of patient enrollment. There is an overlap between the group of patients included in the study and the group of subjects with records of poor quality, because, in the case of 9 patients, only one eye could be evaluated (e.g., due to nystagmus, difficulty with pupil dilation). Cardiovascular risk groups were constructed based on severity from “A” to “C”. Subjects of group “A” were the least affected, while individuals of groups “B” and “C” had already undergone aortic surgery. Taking into account the number of individuals in each group and the need for aortic surgery, subjects of groups “B” and “C” were also analyzed together. DN: dominant negative, HI: haploinsufficient, MFS: Marfan syndrome.

To characterize genotype-phenotype associations, patients were classified according to the type of mutation into HI and DN groups. The amino acid cysteine plays a particularly important role in the structure of the fibrillin-1 protein through the formation of disulfide bridges. Literature and previous own data also confirm that mutations affecting cysteine are associated with more severe aortic involvement [7,19]. Therefore, depending on whether the DN mutation was associated with cysteine elimination or not, two further subgroups were created: DN (-Cys) and DN (non-Cys), respectively. Participants were then grouped according to which cardiovascular risk group they fell into. First, two groups were compared based on whether the patients had previously undergone aortic surgery (operated vs. non-operated subjects). This was followed by a three-group comparison study as follows: individuals without aortic involvement necessitating surgery were classified into group A (there was no aortic dissection, no aortic valve insufficiency, and the diameter of the ascending aorta was less than 45 mm). Group B included subjects with mild cardiovascular involvement who required elective surgery of the ascending aorta, indicated according to the European Society of Cardiology guideline [20]: diameter of the ascending aorta is 45–50 mm OR the sinus of Valsalva is 45–48 mm with grade I-II aortic valve regurgitation AND 2 mm/year increase in size on repeated testing OR a family history of aortic root dissection. Group C included patients with severe cardiovascular involvement who had undergone surgery for annuloaortic ectasia (ascending aorta > 50 mm OR sinus of Valsalva > 48 mm with grade III-IV aortic regurgitation) or type A aortic dissection [21]. The enrollment and classification of patients by variant type and cardiovascular involvement is shown in Fig 1.

### Ophthalmological examination

128 eyes of 64 MFS patients with positive genetic results were examined. The pupil was dilated with 1 drop of tropicamide (5 mg/ml) and 1 drop of phenylephrine (10%) with 30 minutes dilation time. All subjects underwent OCT angiography imaging with the Optovue AngioVue OCT angiography system (software version 2017.1, 7.0 phase upgrade), using split-spectrum amplitude-decorrelation angiography software algorithm (RTVue XR Avanti with AngioVue, Optovue Inc, Fremont, CA, USA) to record 6 × 6 mm OCT angiograms, centered on the macula. 70,000 a-scans per second were acquired with an axial resolution of 5 µm. First, the software extracts a binary image from the OCTA image. It then calculates the vessel density as the percentage of the area in the given region covered by microvascular pixels. The software automatically segments the entire retinal vascular network into full-thickness inner retina, superficial capillary plexus, and deep capillary plexus. The vessel density can thus be derived for each region within a given OCTA segmentation [22,23]. The following parameters were measured with the built-in automated AngioAnalytics software of the OptoVue system: retinal thickness (RT), superficial retinal vessel density (SVD), deep retinal vessel density (DVD) in the whole, foveal, parafoveal, perifoveal areas of the retina, as well as foveal avascular zone (FAZ), perimeter of FAZ at the level of the superficial capillary plexus, and fractal dimension (FD).

In many patients, it was not possible to obtain a well-evaluated record due to difficult pupil dilation or nystagmus. Therefore, images with segmentation defects either at the level of the superficial or deep vascular plexus were excluded from the study. We also excluded images containing artificial defects (double vessel pattern, dark areas from blinking or caused by poor fixation, white line artifacts and vessel ruptures caused by microsaccades). For further analysis, we used the acceptable image quality threshold recommended by Optovue, therefore, only OCT angiograms with a scan quality index of 6 or higher were considered in the analysis.

### Statistical analysis

The distribution of continuous variables was evaluated using the Shapiro-Wilk test and the Kolmogorov-Smirnov test. Given that OCTA parameters often show significant asymmetry between the two sides [23,24], data for both eyes were considered in cases where the images were of sufficient quality. The averages of the data for both eyes were calculated for each patient. For variables with a normal distribution, a two-sample t-test was applied, while variables with a non-normal distribution were tested using the Mann-Whitney-Wilcoxon test. Comparisons between more than two groups were performed using the analysis of variance (ANOVA) test and the corresponding post-hoc test. For categorical variables, comparisons were performed using the χ2 test or the Fisher exact test, according to the number of entries. In all instances, two-sided tests were conducted, with p < 0.05 chosen as the significance level.

## Results

### Baseline characteristics

Based on the quality of the images, 69 eyes of 39 patients were included in the study. For 9 patients, only recordings from one eye were of sufficient quality to be considered for the assessment. Of the 39 patients, 14 (35.9%) were males and 25 (64.1%) were females. The average age was 32.5 ± 13.1 years (from 7 to 55 years). By genetic variant type, 20 (51.3%) HI and 19 (48.7%) DN subjects were examined. DN patients were then further divided into two subgroups according to whether the mutation resulted in cysteine elimination, thus, in addition to the HI group, 9 (23.1%) patients with cysteine elimination [DN (-Cys)] and 10 (25.6%) patients without cysteine elimination [DN (non-Cys)] were compared. The baseline characteristics of the patients did not differ significantly by variant type (Table 1).

**Table 1:** Baseline characteristics of examined patients.

	HI (n = 20)	DN (non-Cys) (n = 10)	DN(-Cys) (n = 9)	Total (n = 39)	p value
**Gender (males:females)**	6:14	4:6	4:5	14:25	0.747
**Age (years)**	33.6 ± 14.3	33.0 ± 18.6	29.9 ± 13.7	32.6 ± 15.0	0.831
**Height (cm)**	180 ± 10.0	181 ± 17.3	182 ± 8.4	181 ± 11.7	0.949
**Lower segment (cm)**	95.7 ± 5.2	96.9 ± 4.6	100.0 ± 7.7	96.8 ± 5.7	0.230
**Arm span (cm)**	188 ± 9.3	190 ± 12.1	183 ± 13.9	187 ± 10.9	0.514
**Foot size**	42.5 ± 2.1	42.4 ± 2.5	43.0 ± 3.7	42.6 ± 2.5	0.964
**Weight (kg)**	63.4 ± 14.6	73.7 ± 27.4	64.3 ± 14.9	66.4 ± 18.9	0.366
**Body mass index (kg/m**^**2**^)	19.4 ± 3.5	22.0 ± 6.9	19.3 ± 3.8	20.1 ± 4.7	0.319
Body surface area (cm*kg)	1.76 ± 0.3	1.87 ± 0.5	1.79 ± 0.2	1.79 ± 0.3	0.688
**USLS**	0.89 ± 0.07	0.89 ± 0.06	0.82 ± 0.07	0.87 ± 0.07	0.072
**ASHR**	1.04 ± 0.04	1.02 ± 0.06	1.01 ± 0.04	1.03 ± 0.04	0.210
**Systemic score**	7.8 ± 2.0	6.5 ± 3.2	8.5 ± 1.6	7.6 ± 2.4	0.185

ASHR: Arm span – Height ratio, USLS: Upper segment – Lower Segment ratio, DN (non-Cys): dominant negative mutation not eliminating a cysteine amino acid, DN (-Cys): dominant negative mutation eliminating a cysteine amino acid, HI: haploinsufficient.

### Genotype-phenotype associations

**Fig 2:**
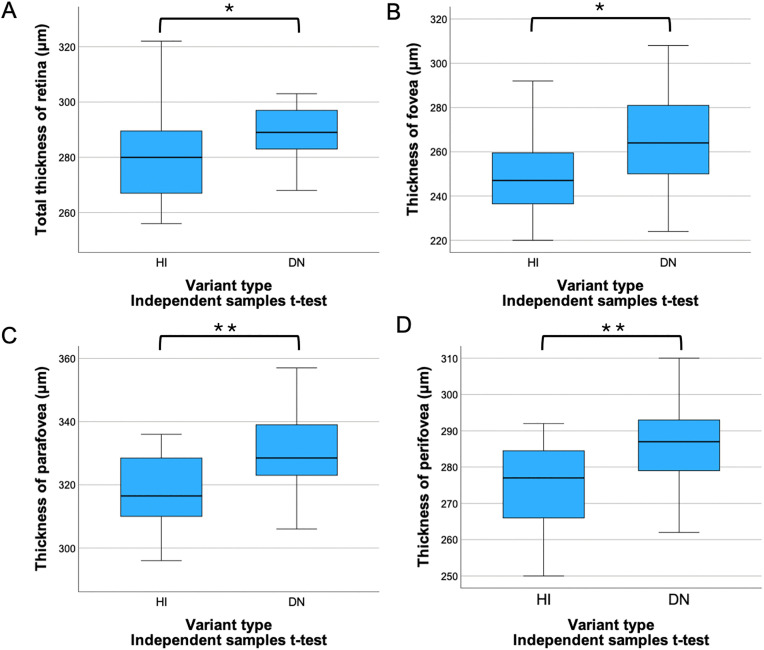
Retinal thickness in different regions by 2 variation types. The mean retinal thickness of the whole retina **(A)**, fovea **(B)**, parafovea **(C)** and perifovea **(D)** of patients with haploinsufficient variants was significantly thinner than that of dominant negative subjects. A Total thickness of retina (µm). B Thickness of fovea (µm). C Thickness of parafovea (µm). D Thickness of perifovea (µm). DN: dominant negative, HI: haploinsufficient, *: p < 0.05, **: p < 0.01.

Comparing the OCTA parameters of the HI and DN groups, the retina of HI individuals was found to be significantly thinner in the total, foveal, parafoveal, and perifoveal regions (t-test p = 0.020, p = 0.047, p = 0.001, p = 0.007, respectively) (Fig 2).

**Fig 3:**
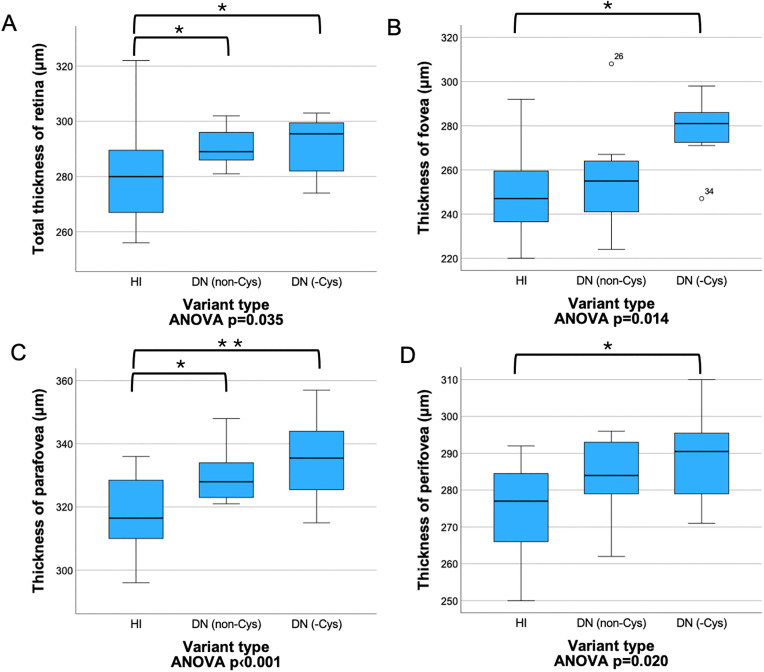
Retinal thickness in different regions by 3 variant types. Retina of patients with haploinsufficient variants was significantly thinner in the entire retina **(A)**, in the fovea **(B)**, parafovea **(C)**, and perifovea **(D)**, compared to DN (-Cys) subjects. This difference was also observed in the total retina (A) and parafovea (C) compared to DN (non-Cys) patients. A Total thickness of retina (µm). B Thickness of fovea (µm). C Thickness of parafovea (µm). D Thickness of perifovea (µm). DN (non-Cys): dominant negative mutation not eliminating a cysteine amino acid, DN (-Cys): dominant negative mutation eliminating a cysteine amino acid, HI: haploinsufficient. Bonferroni post hoc test was applied → *: p < 0.05, **: p < 0.01. (In the analysis of total thickness of retina, the Bonferroni post hoc test showed no significance, therefore the LSD post hoc test was used → *: p < 0.05).

In the 3-group analysis, the retina of HI patients was significantly thinner compared to both DN (non-Cys) and DN (-Cys) subjects in the total (LSD test p = 0.038 and p = 0.032) and parafoveal areas (Bonferroni test p = 0.027 and p = 0.002). In the foveal and perifoveal regions, the retina of HI subjects was also significantly thinner compared to DN (-Cys) patients (Bonferroni test p = 0.011 and p = 0.029) (Fig 3).

No significant differences were found between the groups in terms of SVD, DVD, area and perimeter of FAZ, and FD. Interestingly, there was no significant difference between the cardiovascular risk groups according to the type of mutation [HI vs. DN: p = 0.566; HI vs. DN (non-Cys) vs. DN (-Cys): p = 0.737]. Retinal parameters by 2 and 3 variant types are summarized in Supplementary (S1 and S2 Tables).

### Relationships between retinal blood flow and cardiovascular risk groups

**Fig 4:**
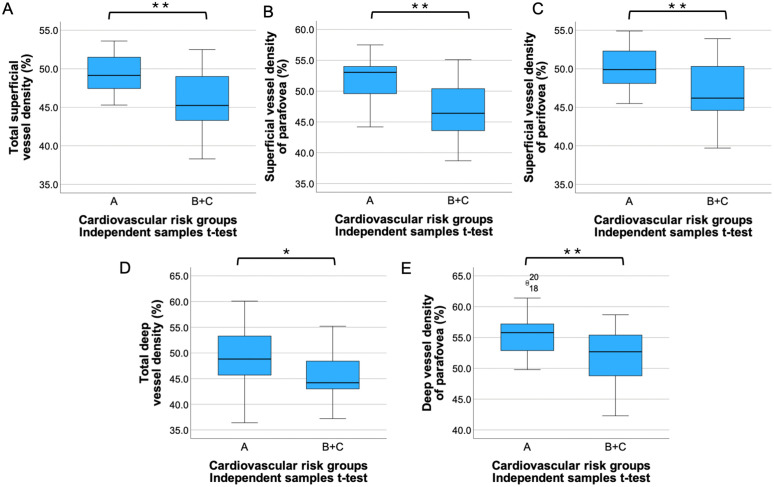
Retinal vessel density by 2 cardiovascular risk groups. Total **(A)**, parafoveal **(B)**, perifoveal superficial vessel density **(C)**, total **(D)** and parafoveal **(E)** deep vessel density were significantly higher in group “A” compared to patients who underwent aortic surgery [group (B + C)]. *: p < 0.05, **: p < 0.01. A Total superficial vessel density (%). B Superficial vessel density of parafovea (%). C Superficial vessel density of perifovea (%). D Total deep vessel density (%). E Deep vessel density of parafovea (%).

Considering cardiovascular risk, total, parafoveal, and perifoveal SVD, total and parafoveal DVD of non-operated subjects were significantly higher than those in operated patients (t-test p = 0.004, p = 0.002, p = 0.007, p = 0.043 and p = 0.008, respectively) (Fig 4, S3 Table).

**Fig 5:**
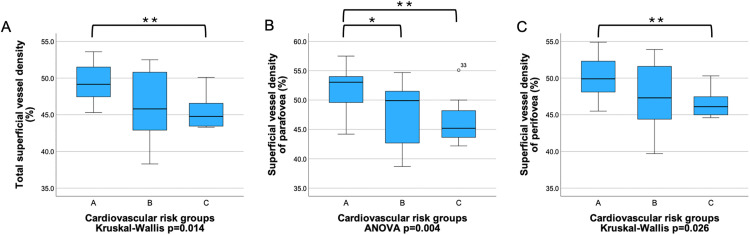
Retinal vessel density by 3 cardiovascular risk groups. Total **(A)**, parafoveal **(B)**, perifoveal superficial vessel density **(C)** were significantly higher in patients in group A compared to the most severely affected cardiovascular group [group C]. Parafoveal superficial vessel density was also significantly higher in group A compared to group B. Bonferroni post hoc test or pairwise comparison was applied → *: p < 0.05, **: p < 0.01. A Total superficial vessel density (%). B Superficial vessel density of parafovea (%). C Superficial vessel density of perifovea (%).

When the operated patients were further divided into two groups (B and C), there was also a significant difference in total, parafoveal, and perifoveal SVD between groups A and C (pairwise comparisons p = 0.005, p = 0.007, and p = 0.009, respectively). In the case of parafoveal SVD, there was also a significant difference between groups A and B (Bonferroni test p = 0.049). The averages and differences of the OCTA parameters of three cardiovascular risk groups (A, B and C) are shown in Fig 5 and S4 Table.

The fractal dimension did not differ significantly between the groups [A vs. (B + C) groups: p = 0.122, A vs. B vs. C groups: p = 0.597].

### Associations between retinal vessel density and age

The age difference between operated and non-operated patients showed weak significance (p = 0.045) with non-operated patients being younger (mean age of non-operated subjects: 27.7 ± 14.6 years, operated patients: 39.5 ± 12.6 years). The average age of members of groups A, B and C did not differ significantly (p = 0.124, mean age of group A: 27.7 ± 14.6 years, group B: 38.4 ± 9.4 years, group C: 40.8 ± 16.1 years). A downward trend in total SVD with advancing age was observed in our study, the annual decrease being 0.10% in the non-operated group and 0.12% in operated subjects (S1 Fig). Total SVD and age values were not correlated (p = 0.068, r = −0.30) (S2 Fig).

## Discussion

The most widely studied genetic disorder associated with *FBN1* gene mutations is MFS [25]. Fibrillin-1 is the major component of the extracellular microfibrils, providing elasticity and structural support to the connective tissue [26]. *FBN1* mutations characteristic to MFS can occur along the entire length of the gene. Their common feature is that they cause damage to the elastic fibres, disrupting the stability of the extracellular matrix [27]. Diagnostic evaluation of MFS is inevitably complex, as the symptoms of affected individuals are highly variable, many are age-dependent, and the differential diagnosis is extensive [28]. Early detection of the disease is essential in preventing acute vascular events. Accordingly, it is of paramount significance to gain a more accurate understanding of genotype-phenotype relationships and the co-occurrence of life-threatening cardiovascular manifestations with other features, such as ophthalmic involvement.

### Genotype-phenotype associations

In recent years, increasing efforts were made to elucidate genotype-phenotype associations in MFS patients, but the ophthalmological aspects are still poorly understood. Several working groups have observed associations, but these mostly concern the anterior segment of the eye. One of the best-known genotype-phenotype associations is that DN patients, especially those with a mutation affecting the amino acid cysteine, have a higher incidence of EL and are expected to develop more severe ocular symptoms [9,13]. Moreover, myopia occurs earlier in DN variants affecting the cysteine amino acid [29]. Guo et al. also described higher corneal astigmatism and severe lens dislocation in subjects with missense variants compared to premature termination codon mutations [30]. Another study examining EL patients reported thinner central corneal thickness in patients with HI mutations [31].

In contrast to the growing body of evidence supporting genotype-phenotype correlations involving anterior segment parameters, to the best of our knowledge, no previous publication has reported relationships between variant type and OCTA parameters. Our working group is the first to observe that the retina of HI patients is significantly thinner in the total, foveal, parafoveal, and perifoveal regions than that of DN patients. Due to the more severe ocular manifestations of DN mutations eliminating cysteine, we compared the OCTA parameters by classifying DN (-Cys) patients into a separate group. The retina of HI patients was significantly thinner than that of both DN (non-Cys) and DN (-Cys) subjects in the total, parafoveal area, and thinner than that of DN (-Cys) patients in the fovea and perifovea. An important clinical relevance of these findings could be that OCTA examination is highly repeatable and reproducible, easy to perform, and does not involve complications. The retinal thickness values can be retrieved from the device immediately after the image is taken, eliminating the need for manual measurements. OCTA parameters could, therefore, serve as promising biomarkers for both diagnosis and prognosis in MFS [18,32].

### Relationship between retinal blood flow and cardiovascular manifestations

Several studies have reported that the severity of cardiovascular parameters in MFS is associated with impairment of retinal blood flow [14,18,32], and the OCTA parameters were mostly compared to specific cardiovascular metrics. However, there was a lack of specific OCTA parameters that could be clearly associated with higher cardiovascular risk.

Decreased expression of fibrillin-1 in the aortic wall leads to disruption of the extracellular matrix integrity. The aortic media thus exhibits degenerative features such as generalized medial degeneration, fragmentation and loss of elastic fibers. Furthermore, the ascending aortic wall is characterized by less mature vascular smooth muscle cells in MFS, leading to impaired vascular wall contractility. *FBN1* mutations reduce the sequestration of transforming growth factor-beta (TGF-β) in the extracellular matrix and also lead to increased angiotensin II receptor signaling, which enhances TGF-β activity [2,33,34]. The latter induces increased apoptosis, causing the loss of smooth muscle cell nuclei. These cells also play a key role in the aorta, and their destruction by apoptosis leads to immaturity of the aortic wall in subjects with MFS [33]. Fibrillin-1 is also the most common component of elastic microfilaments in the human eye. Its regulatory role in microvascular flow in the retinal vascular system is not yet fully understood [18]. It is presumed that the apoptosis-induced destruction of vascular smooth muscle cells may also occur in the retinal vascular network. This phenomenon may explain changes in VD as measured by OCTA [32].

In our study population, total, parafoveal, perifoveal SVD and total, parafoveal DVD were significantly lower in the operated group. In a three-way comparison, total, parafoveal and perifoveal SVD were also significantly lower in group C compared to group A. Significant difference was observed in terms of parafoveal SVD, with group B having lower values than group A. Our results suggest that SVD (but also DVD) measurements in different retinal regions can provide useful information for monitoring MFS patients and assessing their cardiovascular risk. Our observations are supported by several published works. According to Di Marino et al., there was a negative correlation between left ventricular diameter and VD of the superficial and deep plexuses, as well as between size of the ascending aorta and foveal choriocapillary VD. An increased thickness of posterior wall and interventricular septum were also associated with lower VD in both plexuses [32]. Due to its correlation with cardiovascular involvement, VD measurement may be a promising biomarker. Findings of Chen and colleagues also support the importance of measuring SVD, as they identified significantly reduced SVD and FAZ circulatory index in children with MFS compared to the control group. Moreover, they found a correlation between changes in microvascular flow and visual acuity, central macular thickness, aortic diameter, and left ventricular ejection fraction. According to their results, patients with larger ascending aorta diameter and left ventricular dysfunction also exhibit retinal blood flow disturbances, suggesting that patients with MFS have retinal vascular abnormalities in addition to aortopathy [18]. According to the findings of Rezar-Dreindl et al., retinal microvascular changes can be observed in patients with lens displacement and cardiovascular symptoms. An increase in area of FAZ was observed in conjunction with a decrease in SVD. In addition, a weak positive correlation was observed between the severity of systemic vascular disease and the area of FAZ, as well as a negative correlation with central vessel density [3]. In our study, the area of FAZ was also examined, but there was no significant difference between patients classified into different cardiovascular risk groups.

It has been previously reported in the literature that retinal VD decreases with age [35–37]. This raises the question of whether the differences in SVD observed in our study among cardiovascular risk groups are due to age differences. The difference in age between operated and non-operated patients showed weak significance (p = 0.045), but there was no significant difference between the average ages of groups A, B, and C (p = 0.124). Based on the study by Iafe and colleagues, the annual decrease in SVD was found to be 0.24% with advancing age in normal subjects when examining 6 × 6 mm angiograms [37]. Although a downward trend in total SVD with advancing age was observed in our study, the annual decrease was less compared to literature data in the group of both the non-operated (0.10%) and the operated (0.12%) subjects, without any substantial difference between these two groups (S1 Fig). The correlation between total SVD and age found no relationship between the two parameters (p = 0.068, r = −0.30) (S2 Fig). This suggests that the significantly lower SVD values in patients with higher cardiovascular risk were not due to age differences.

FD is an increasingly studied retinal geometric parameter that serves as a global index for the quantitative assessment of the geometric complexity of the retinal vascular network, including all branching patterns. It can be an effective indicator for the early detection of microvascular diseases, even before the clinical symptoms of retinopathy appear. According to the literature, low FD values indicate less dense branching patterns, which may be associated with cardiovascular mortality risk and advanced stages of diabetic retinopathy [38]. However, no significant difference was observed in operated patients compared to non-operated subjects in our study.

Limitations of the study include the absence of a control group and the relatively small number of patients involved. Many MFS subjects had to be excluded due to the difficulty to obtain images of sufficient quality. The most common reasons for this were nystagmus and pupils that were difficult to dilate. In addition to reporting greater photophobia [39], literature reports that MFS patients have a smaller maximum pupil diameter, slower pupil constriction, and longer pupil re-dilatation times compared to the control group [40]. However, given the rarity of MFS, the results of our study may provide valuable insights into genotype-phenotype relationships and aid in the identification of patients at elevated risk of cardiovascular disease.

In this analysis, to the best of our knowledge, we were the first to show that the retina of HI patients is significantly thinner in several regions compared to DN (non-Cys) and DN (-Cys) patients. In line with the previous reports, we confirmed that SVD is significantly lower in patients with a higher risk of cardiovascular manifestation. All these findings support the importance of OCTA examination in the diagnosis and follow-up of MFS patients. Larger studies are needed to further understand the genotype-phenotype correlations, to precisely determine how the severity of systemic, and retinal vascular changes are associated, and to be able to identify robust prognostic OCTA markers.

## Supporting information

S1 Table Retinal parameters by 2 variant types. DN: dominant negative, FAZ: foveal avascular zone, HI: haploinsufficient, *: p < 0.05.
(PDF)

S2 Table Retinal parameters by 3 variant types. DN (non-Cys): dominant negative mutation not eliminating a cysteine amino acid, DN (-Cys): dominant negative mutation eliminating cysteine, FAZ: foveal avascular zone, HI: haploinsufficient, *: p < 0.05.
(PDF)

S3 Table Retinal parameters by 2 cardiovascular groups. Cardiovascular risk groups were divided into categories from “A” to “C” based on the severity of the disease. Subjects in group “A” were the least affected, while those in groups “B” and “C” had already undergone aortic surgery. Taking into consideration the number of subjects in each group and the need for aortic surgery, subjects in groups “B” and “C” were examined together. FAZ: foveal avascular zone, *: p < 0.05.
(PDF)

S4 Table Retinal parameters by 3 cardiovascular groups. Based on the severity of the disease, cardiovascular risk groups were assigned to categories “A” through “C”. Subjects in group “A” were the least severely affected, while those in groups “B” and “C” had already undergone aortic surgery. FAZ: foveal avascular zone, *: p < 0.05.
(PDF)

S1 Fig A linear regression model of age and total superficial vessel density. Although total superficial vessel density decreased with age in the studied population (especially in patients who had undergone aortic surgery), this reduction was not as marked as it would be expected from the literature (Iafe NA et al., Invest Ophthalmol Vis Sci, 2016.). Cardiovascular risk groups were constructed by severity from “A” to “C”. Subjects of group “A” were the least affected, while individuals of groups “B” and “C” had undergone aortic surgery.
(PDF)

S2 Fig Correlation between age and total superficial vessel density. Data of age and total superficial vessel density were not correlated [p = 0.068, r = −0.30], thus the significantly lower total superficial vessel density of patients who underwent aortic surgery cannot be explained by the higher age of this group.
(PDF)

## Abbreviations

DN: dominant negative
DN (-Cys): dominant negative mutation eliminating a cysteine amino acid
DN (non-Cys): dominant negative mutation not eliminating a cysteine amino acid
DVD: deep vessel density
EL: ectopia lentis
FAZ: foveal avascular zone
*FBN1*: fibrillin-1 gene
FD: fractal dimension
HI: haploinsufficient
MFS: Marfan syndrome
OCT: optical coherence tomography
OCTA: optical coherence tomography angiography
RT: retinal thickness
SVD: superficial vessel density
TGF-β: transforming growth factor-beta
VD: vessel density
