# Supplementary material for: Optical coherence tomography angiography parameters in Marfan syndrome: Genetic determinants and associations with cardiovascular manifestations
Source: PLoS One. 2026 Apr 24;21(4):e0347666. doi: 10.1371/journal.pone.0347666 (PMC13108799; doi:10.1371/journal.pone.0347666)
Supplement: S1 Table — DN: dominant negative, FAZ: foveal avascular zone, HI: haploinsufficient, *: p < 0.05. (PDF) [file pone.0347666.s001.pdf]

TABLE S1. Retinal parameters by 2 variant types					
		**HI (n=20, 35 eyes)**	**DN (n=19, 34 eyes)**	**Total (n=39, 69 eyes)**	**p value**
**Retinal thickness (μm)**	Total	280±15.8	285±17.4	283±16.7	*0.020**
	Fovea	251±17.9	263±5.9	257±22.8	*0.047**
	Parafovea	317±12.2	327±17.3	322±15.7	*0.001**
	Perifovea	275±12.7	283±5.9	279±14.8	*0.007**
**Superficial vessel density (%)**	Total	47.2±3.8	48.1±4.9	47.6±4.4	0.748
	Fovea	20.6±7.7	20.3±6.9	20.4±7.3	0.554
	Parafovea	49.1±6.1	49.7±6.7	49.4±6.3	0.661
	Perifovea	48.2±3.6	48.9±4.7	48.7±4.2	0.564
**Deep vessel density (%)**	Total	47.7±7.0	48.2±6.4	47.9±6.7	0.599
	Fovea	39.2±7.1	37.3±8.4	38.2±7.8	0.951
	Parafovea	53.4±6.1	53.8±5.2	53.6±5.6	0.640
	Perifovea	48.9±7.7	49.2±7.4	49.0±7.5	0.971
**FAZ (mm^2)**		0.241±0.09	0.260±0.11	0.251±0.10	0.552
**Perimeter of FAZ (mm)**		1.90±0.39	1.97±0.47	1.93±0.43	0.367
**Fractal dimension**		52.3±5.5	50.6±7.1	51.5±6.4	0.321

DN: dominant negative, FAZ: foveal avascular zone, HI: haploinsufficient, *: $p < 0.05$.
