# Supplementary material for: Optical coherence tomography angiography parameters in Marfan syndrome: Genetic determinants and associations with cardiovascular manifestations
Source: PLoS One. 2026 Apr 24;21(4):e0347666. doi: 10.1371/journal.pone.0347666 (PMC13108799; doi:10.1371/journal.pone.0347666)
Supplement: S2 Table — DN (non-Cys): dominant negative mutation not eliminating a cysteine amino acid, DN (-Cys): dominant negative mutation eliminating cysteine, FAZ: foveal avascular zone, HI: haploinsufficient, *: p < 0.05. (PDF) [file pone.0347666.s002.pdf]

TABLE S2. Retinal parameters by 3 variant types						
		**HI (n=20, 35 eyes)**	**DN (non-Cys) (n=10, 19 eyes)**	**DN (-Cys) (n=9, 15 eyes)**	**Total (n=39, 69 eyes)**	**p value**
**Retinal thickness (μm)**	Total	280±15.8	287±9.2	283±24.3	283±16.7	0.035*
	Fovea	251±17.9	253±19.2	274±29.0	257±22.8	0.014*
	Parafovea	317±12.2	326±10.4	329±23.7	322±15.7	<0.001*
	Perifovea	275±12.7	283±10.1	282±21.4	279±14.8	0.020*
**Superficial vessel density (%)**	Total	47.2±3.8	48.9±4.3	47.1±5.5	47.6±4.4	0.578
	Fovea	20.6±7.7	19.1±5.4	21.8±8.4	20.4±7.3	0.221
	Parafovea	49.1±6.1	50.5±5.8	48.8±7.8	49.4±6.3	0.825
	Perifovea	48.2±3.6	49.9±4.3	48.1±5.2	48.7±4.2	0.558
**Deep vessel density (%)**	Total	47.7±7.0	48.1±6.5	48.3±6.6	47.9±6.7	0.992
	Fovea	39.2±7.1	36.9±6.7	37.8±10.4	38.2±7.8	0.458
	Parafovea	53.4±6.1	54.0±4.7	53.5±5.9	53.6±5.6	0.912
	Perifovea	48.9±7.7	49.2±7.6	49.1±7.5	49.0±7.5	0.977
**FAZ (mm^2)**		0.241±0.09	0.262±0.10	0.258±0.13	0.251±0.10	0.468
**Perimeter of FAZ (mm)**		1.90±0.39	1.97±0.45	1.96±0.51	1.93±0.43	0.896
**Fractal dimension**		52.2±5.5	49.5±8.0	52.1±5.7	51.5±6.4	0.597

DN (non-Cys): dominant negative mutation not eliminating a cysteine amino acid, DN (-Cys): dominant negative mutation eliminating cysteine, FAZ: foveal avascular zone, HI: haploinsufficient, *: $p < 0.05$.
