# Supplementary material for: Optical coherence tomography angiography parameters in Marfan syndrome: Genetic determinants and associations with cardiovascular manifestations
Source: PLoS One. 2026 Apr 24;21(4):e0347666. doi: 10.1371/journal.pone.0347666 (PMC13108799; doi:10.1371/journal.pone.0347666)
Supplement: S3 Table — Cardiovascular risk groups were divided into categories from “A” to “C” based on the severity of the disease. Subjects in group “A” were the least affected, while those in groups “B” and “C” had already undergone aortic surgery. Taking into consideration the number of subjects in each group and the need for aortic surgery, subjects in groups “B” and “C” were examined together. FAZ: foveal avascular zone, *: p < 0.05. (PDF) [file pone.0347666.s003.pdf]

TABLE S3. Retinal parameters by 2 cardiovascular groups					
		**Group A (n=22, 39 eyes)**	**Group (B+C) (n=17, 30 eyes)**	**Total (n=39, 69 eyes)**	**p value**
**Retinal thickness (μm)**	Total	284±14.2	281±19.7	283±16.7	0.769
	Fovea	255±23.7	258±21.9	257±22.8	0.913
	Parafovea	321±11.7	323±19.9	322±15.7	0.408
	Perifovea	279±11.8	278±18.0	279±14.8	0.474
**Superficial vessel density (%)**	Total	48.7±3.8	46.2±4.7	47.6±4.4	*0.004**
	Fovea	21.3±7.6	19.4±6.8	20.4±7.3	0.517
	Parafovea	50.9±6.2	47.4±6.1	49.4±6.3	*0.002**
	Perifovea	49.8±3.5	47.3±4.7	48.7±4.2	*0.007**
**Deep vessel density (%)**	Total	49.5±6.7	45.8±6.1	47.9±6.7	*0.043**
	Fovea	38.8±7.3	37.5±8.4	38.2±7.8	0.778
	Parafovea	55.0±5.7	51.7±5.0	53.6±5.6	*0.008**
	Perifovea	50.9±7.7	46.7±6.7	49.0±7.5	0.052
**FAZ (mm^2)**		0.249±0.10	0.252±0.09	0.251±0.10	0.439
**Perimeter of FAZ (mm)**		1.91±0.48	1.95±0.36	1.93±0.43	0.580
**Fractal dimension**		52.7±5.9	49.9±6.7	51.5±6.4	0.122

Cardiovascular risk groups were divided into categories from "A" to "C" based on the severity of the disease. Subjects in group “A” were the least affected, while those in groups “B” and “C” had already undergone aortic surgery. Taking into consideration the number of subjects in each group and the need for aortic surgery, subjects in groups “B” and “C” were examined together.

FAZ: foveal avascular zone, *: $p < 0.05$.
