# Supplementary material for: Optical coherence tomography angiography parameters in Marfan syndrome: Genetic determinants and associations with cardiovascular manifestations
Source: PLoS One. 2026 Apr 24;21(4):e0347666. doi: 10.1371/journal.pone.0347666 (PMC13108799; doi:10.1371/journal.pone.0347666)
Supplement: S4 Table — Based on the severity of the disease, cardiovascular risk groups were assigned to categories “A” through “C”. Subjects in group “A” were the least severely affected, while those in groups “B” and “C” had already undergone aortic surgery. FAZ: foveal avascular zone, *: p < 0.05. (PDF) [file pone.0347666.s004.pdf]

TABLE S4. Retinal parameters by 3 cardiovascular groups						
		**Group A (n=22, 39 eyes)**	**Group B (n=9, 17 eyes)**	**Group C (n=8, 13 eyes)**	**Total (n=39, 69 eyes)**	**p value**
**Retinal thickness (μm)**	Total	284±14.2	280±23.0	283±14.9	283±16.7	0.759
	Fovea	255±23.7	257±23.3	260±20.6	257±22.8	0.566
	Parafovea	321±11.7	322±23.4	324±14.9	322±15.7	0.813
	Perifovea	279±11.8	279±20.1	279±15.7	279±14.8	0.877
**Superficial vessel density (%)**	Total	48.7±3.8	46.6±5.6	45.8±3.3	47.6±4.4	*0.014**
	Fovea	21.3±7.6	19.3±7.8	19.4±5.4	20.4±7.3	0.753
	Parafovea	50.9±6.2	47.9±6.8	46.9±5.2	49.4±6.3	*0.004**
	Perifovea	49.8±3.5	47.7±5.6	46.7±3.1	48.7±4.2	*0.026**
**Deep vessel density (%)**	Total	49.5±6.7	45.5±7.2	46.3±4.5	47.9±6.7	0.130
	Fovea	38.8±7.3	37.0±9.9	38.2±6.3	38.2±7.8	0.716
	Parafovea	55.0±5.7	51.0±5.8	52.7±3.9	53.6±5.6	0.217
	Perifovea	50.9±7.7	46.5±7.9	47.0±5.0	49.0±7.5	0.154
**FAZ (mm^2)**		0.249±0.10	0.269±0.11	0.230±0.07	0.251±0.10	0.636
**Perimeter of FAZ (mm)**		1.91±0.48	1.99±0.41	1.89±0.29	1.93±0.43	0.755
**Fractal dimension**		52.7±5.9	49.8±7.3	50.0±6.5	51.5±6.4	0.285

Based on the severity of the disease, cardiovascular risk groups were assigned to categories “A” through “C”. Subjects in group “A” were the least severely affected, while those in groups “B” and “C” had already undergone aortic surgery.

FAZ: foveal avascular zone, *: $p < 0.05$.
