# Supplementary material for: Optical coherence tomography angiography parameters in Marfan syndrome: Genetic determinants and associations with cardiovascular manifestations
Source: PLoS One. 2026 Apr 24;21(4):e0347666. doi: 10.1371/journal.pone.0347666 (PMC13108799; doi:10.1371/journal.pone.0347666)
Supplement: S1 Fig — Although total superficial vessel density decreased with age in the studied population (especially in patients who had undergone aortic surgery), this reduction was not as marked as it would be expected from the literature (Iafe NA et al., Invest Ophthalmol Vis Sci, 2016.). Cardiovascular risk groups were constructed by severity from “A” to “C”. Subjects of group “A” were the least affected, while individuals of groups “B” and “C” had undergone aortic surgery. (PDF) [file pone.0347666.s005.pdf]

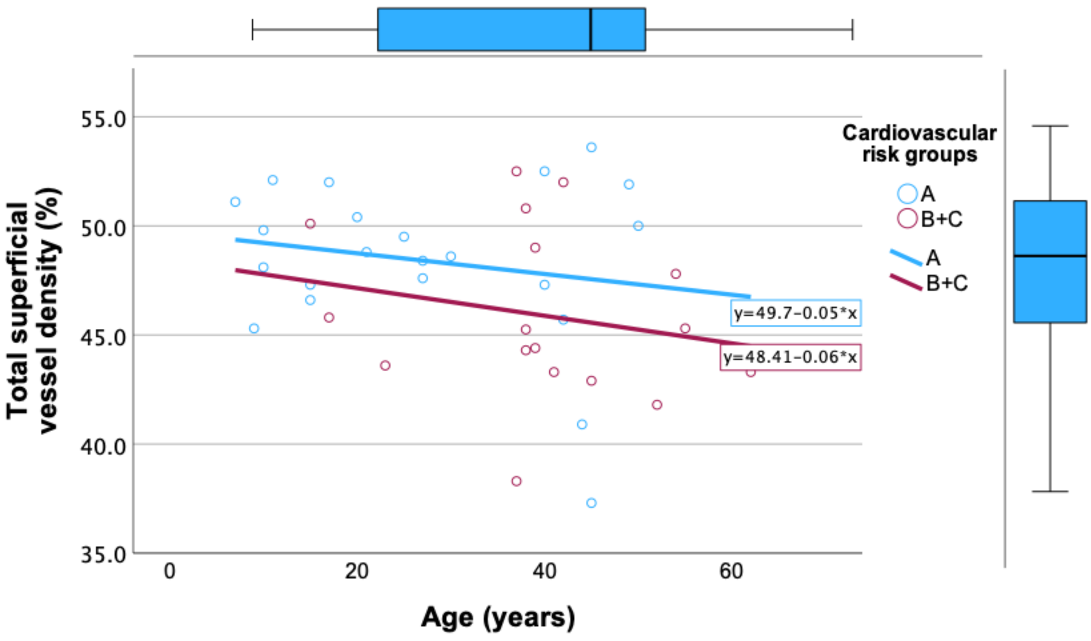

FIGURE S1. A linear regression model of age and total superficial vessel density.

Although total superficial vessel density decreased with age in the studied population (especially in patients who had undergone aortic surgery), this reduction was not as marked as it would be expected from the literature (Iafe NA et al., Invest Ophthalmol Vis Sci, 2016.). Cardiovascular risk groups were constructed by severity from “A” to “C”. Subjects of group “A” were the least affected, while individuals of groups “B” and “C” had undergone aortic surgery.
