# Supplementary material for: Optical coherence tomography angiography parameters in Marfan syndrome: Genetic determinants and associations with cardiovascular manifestations
Source: PLoS One. 2026 Apr 24;21(4):e0347666. doi: 10.1371/journal.pone.0347666 (PMC13108799; doi:10.1371/journal.pone.0347666)
Supplement: S2 Fig — Data of age and total superficial vessel density were not correlated [p = 0.068, r = −0.30], thus the significantly lower total superficial vessel density of patients who underwent aortic surgery cannot be explained by the higher age of this group. (PDF) [file pone.0347666.s006.pdf]

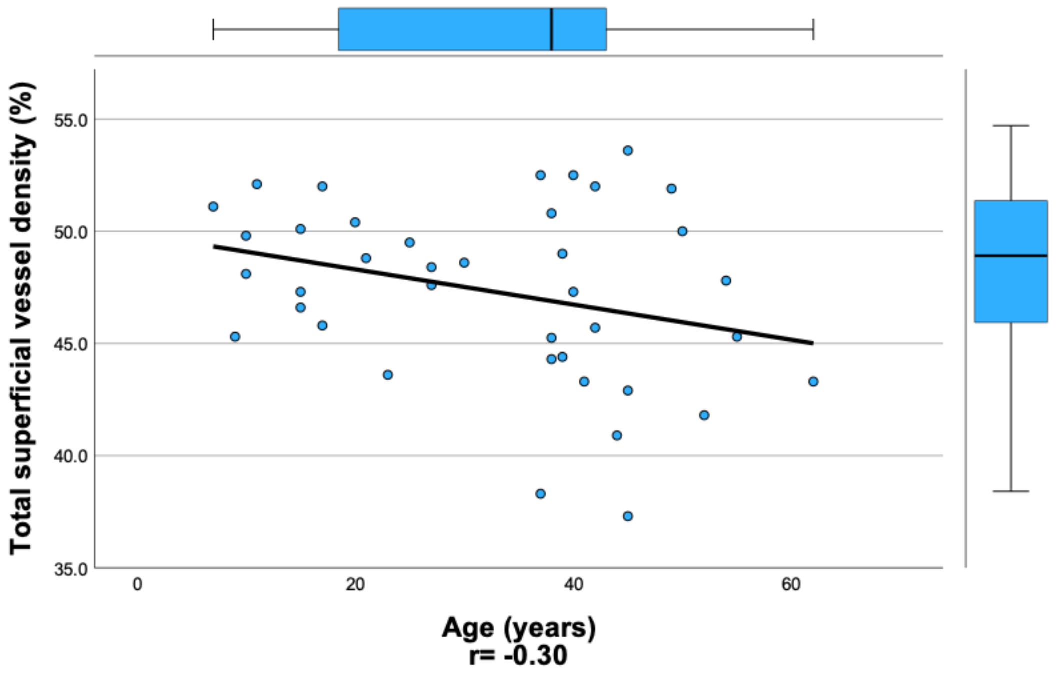

FIGURE S2. Correlation between age and total superficial vessel density.

Data of age and total superficial vessel density were not correlated [p=0.068, r= -0.30], thus the significantly lower total superficial vessel density of patients who underwent aortic surgery cannot be explained by the higher age of this group.
